# A Coalitional Distributed Model Predictive Control Perspective for a Cyber-Physical Multi-Agent Application

**DOI:** 10.3390/s21124041

**Published:** 2021-06-11

**Authors:** Anca Maxim, Constantin-Florin Caruntu

**Affiliations:** Department of Automatic Control and Applied Informatics, “Gheorghe Asachi” Technical University of Iasi, 700050 Iasi, Romania; anca.maxim@ac.tuiasi.ro

**Keywords:** coalitional model predictive control, distributed model-predictive control, multi-agent systems, closed-loop stability

## Abstract

Following the current technological development and informational advancement, more and more physical systems have become interconnected and linked via communication networks. The objective of this work is the development of a Coalitional Distributed Model Predictive Control (C- DMPC) strategy suitable for controlling cyber-physical, multi-agent systems. The motivation behind this endeavour is to design a novel algorithm with a flexible control architecture by combining the advantages of classical DMPC with Coalitional MPC. The simulation results were achieved using a test scenario composed of four dynamically coupled sub-systems, connected through an unidirectional communication topology. The obtained results illustrate that, when the feasibility of the local optimization problem is lost, forming a coalition between neighbouring agents solves this shortcoming and maintains the functionality of the entire system. These findings successfully prove the efficiency and performance of the proposed coalitional DMPC method.

## 1. Introduction

Presently, manifold systems are modular, interconnected and have a cyber-physical setup, meaning they can be viewed as coupled physical sub-systems, which are connected via communication networks [1,2,3,4,5]. For such processes, Distributed Model Predictive Control (DMPC) is a reliable control solution that uses local controllers that compute the control action using both (i) the local information derived from specific sensors and (ii) coupling data received/transmitted using the communication network [6].

As recent studies attest, the DMPC strategy was successfully applied on multi-agent systems in varying applications, such as formation control of autonomous surface and aerial vehicles [7], leader–follower platoons [8,9], traffic signal control [10], temperature regulation systems [11], battery energy storage systems [12] and microgrids [13,14]. In [15], a DMPC strategy for multi-agent systems based on error upper bounds is provided. This criterion is used in a min–max optimization of the cost function to minimize the communication between neighbouring agents. An event-triggered synchronous DMPC for multi-agent systems is introduced in [16]. The method is tailored for dynamically decoupled sub-systems, coupled through a cost function. An event-triggered mechanism designed using the forward difference of the cost function is deployed to activate the local optimization problem at each sampling time; otherwise the agents use the solutions computed in the previous sampling period. In [17], a DMPC to reach consensus for time-varying, multi-agent systems is proposed. The consensus DMPC algorithm is designed for heterogeneous, time-varying decoupled sub-systems, connected uni-directionally with a coupled cost function.

In all the research mentioned above, regardless of the application or the methodology details, one key feature is noticeable, namely that the architecture of both sub-systems and agents (i.e., local controllers) is fixed. The latter is predefined in the initialization phase of the control design, based on the sensors placements and interconnection between the local sub-systems. Therefore, the configuration of the DMPC neighbourhoods (i.e., groups of local sub-systems that are interconnected either dynamically or through cost functions or constraints) is established and predefined [18].

To overcome this shortcoming, a new approach emerged from cooperative game theory framework named Coalitional Control was introduced, with the following characteristics [19,20]: (i) the topology of the communication links between agents is flexible (i.e., links can be enabled or disabled when necessary), (ii) the control strategy encourages the agents to group in cooperative clusters called coalitions (to reduce the communication burden), and (iii) the communication links between agents that ensure their cooperation are weighted and introduce supplementary costs in the cost functions when activated.

Using this foundation, a Coalitional Model Predictive Control (C-MPC) strategy was developed and applied on different applications such as cellular networks in [21,22] or an eight-coupled tank process [23]. Thus, the agents charged with controlling the local sub-systems can form coalitions depending on the activation of the communication links between them. Several topologies can be derived, starting from the default one, which is the decentralized MPC strategy (i.e., with no communication between agents), to the most complex one. described as centralized MPC (i.e., in which all the communication links are active). In between, there are the coalitions between several agents in a neighbourhood, while the remaining ones work independently. In [24], details regarding the feasible regions for tube-based MPC controllers are discussed. The coalitions are associated with different partitions of a large-scale system (i.e., several sub-systems can be joined in a single entity), and their feasible region is analysed.

The main contribution of this work is the development of a novel perspective of the DMPC algorithm, called Coalitional DMPC (C-DMPC), which combines both the advantages and features of classical DMPC strategy with the characteristics of Coalitional MPC. Hence, the envisioned solution is to dynamically reshape the controller network by merging some of the agents within a neighbourhood into coalitions when needed. The advantages of this approach are shown when, due to various reasons (e.g., reference changes, unknown disturbances, etc.), one or more local optimal solutions become infeasible. In this case, to maintain the feasibility and functionality of the interconnected cyber-physical multi-agent system, the agents will decide to form a coalition. This means that inside a coalition, the sub-systems become a single entity and the controllers aggregate and solve a cooperative optimization problem (i.e., a global cost function is minimized) [25].

The main difference of our approach with respect to the cited coalitional literature is that the default topology is a non-cooperative DMPC (i.e., each agent minimizes a local cost function, using received information from its neighbours) [26]. This means that when a coalition occurs, the remaining agents outside the coalitions are not independent but retain their previous status and solve a non-cooperative optimization problem. Thus, depending on the topology, it is possible that the coalition must exchange information with its neighbours (if not all the agents inside a neighbourhood are merged into the coalition). Since all the agents start as non-cooperative players, they use the communication network to share relevant data, according to their coupling within neighbourhoods, and all pertinent communication links are activated and not weighted.

Another key difference is our proposed merging procedure, which selects the agents that will form a coalition. This is done at each agent level, without using a hierarchical supervisory layer. Moreover, when the local optimization problem becomes unfeasible (due to the coupling information), the coalition is activated. Furthermore, two simplified versions of this method with different agent merging procedures were published in [27,28]. In [27], each agent considers that the coupling information received from the neighboring agents is an uncertainty in the local nominal model. When a predefined threshold value for the local uncertainty level is crossed, than a coalition between the agents is formed. The further development of this idea is given in [28], in which the coalition between the agents is formed, when the local optimization problems become infeasible due to the received uncertainty level.

With respect to our previous papers, the method proposed in the current work has significant improvements, such as the following: (i) the network topology is tailored for in-chain coupled sub-systems, with unidirectional communication links; (ii) a more realistic academic example is used for simulation tests, with four heterogeneous sub-systems dynamically coupled through the inputs; (iii) each sub-system model is augmented with an additional state defined as the integral of control error to ensure a non-zero reference tracking; and (iv) multiple coalitions between agents can be simultaneously active at each sampling time.

The remaining of this paper is structured as follows: Section 2 presents the problem formulation and details the proposed method, whereas the simulation configuration, results, and discussions are provided in Section 3. The conclusions of this work and future work plans are addressed in Section 4.

## 2. Problem Formulation

**Figure 1:**
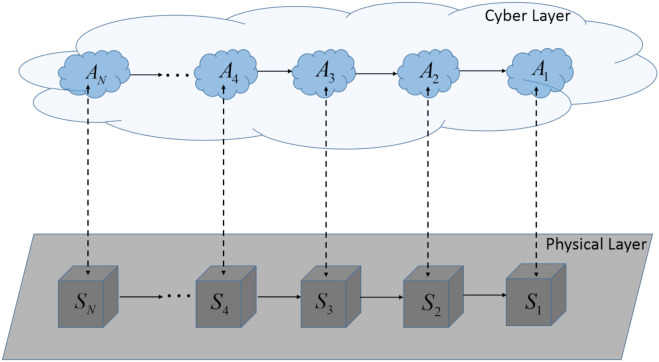
Schematic diagram of a cyber-physical multi-agent system (CP-MAS).

A cyber-physical multi-agent system (CP-MAS), as depicted in Figure 1, is composed of *N* interconnected cyber-physical sub-systems (CPsS). Each CPsS is defined by the pair ($S_{i}$, $A_{i}$), ∀i∈N, where N denotes the set {1,…,N}⊆N, with N∈N the number of sub-systems and N the set of natural numbers. The physical part of the CPsS is denoted with $S_{i}$, whereas the cyber part of the CPsS is denoted with $A_{i}$ and represents the corresponding local controller or agent. All the interconnected sub-systems $S_{i}$ form the physical layer (depicted with grey colour), while the cyber layer (depicted with blue colour) is composed of all the agents and the communication networks.

Let each sub-system $S_{i}$ be defined by the following model:

(1) $$\begin{array}{ccc} x_{p}^{i}\left(k+1\right) & = & A_{p}^{i,i}x_{p}^{i}\left(k\right)+B_{p}^{i,i}u_{p}^{i}\left(k\right)+w_{p}^{i}\left(k\right)\\ w_{p}^{i}\left(k\right) & = & B_{p}^{i,i-1}u_{p}^{i-1}\left(k\right)\\ y_{p}^{i}\left(k\right) & = & C_{p}^{i}x_{p}^{i}\left(k\right),\;\forall i\in N \end{array}$$

with the notations for the state $x_{p}^{i}\in R^{n_{i}}$, input $u_{p}^{i}\in R^{m_{i}}$, input uncertainty $w_{p}^{i}\in R^{p_{i}}$ and output $y_{p}^{i}\in R^{q_{i}}$. $A_{p}^{i,i},\;B_{p}^{i,i},\;B_{p}^{i,i-1}$ and $C_{p}^{i}$ are matrices with adequate dimensions. $n_{i}$, $m_{i}$, $p_{i}$ and $q_{i}$ are the number of states, inputs, input uncertainties and outputs, respectively. R denotes the set of real numbers. Note that, $u_{p}^{i-1}\in R^{m_{i}}$ denotes the input signal received from the predecessor sub-system with index i−1.

Note that (1) defines a model in which the input-coupling information $w_{p}^{i}$ is considered an uncertainty in the nominal model. Moreover, all the sub-systems $S_{i}$, ∀i∈N, are in a chain architecture, and for sub-system indexed *i*, the information is received through an unidirectional link from its predecessor and neighbour, defined as the sub-system with index i−1.

To ensure that the reference tracking control problem has a zero error in stationary regime, the state vector $x_{p}^{i}$, ∀i∈N, from (1) is extended with an additional state ${\bar{x}}_{p}^{i}$ defined as integral of the control error, using the following definition [29,30]:

(2) $${\bar{x}}_{p}^{i}\left(k+1\right)={\bar{x}}_{p}^{i}\left(k\right)-C_{p}^{i}x_{p}^{i}\left(k\right)+r_{i}\left(k\right),$$

obtaining

(3) $$\begin{array}{ccc} \underset{x_{i}\left(k+1\right)}{\underset{︸}{\left[\begin{array}{c} x_{p}^{i}\left(k+1\right)\\ {\bar{x}}_{p}^{i}\left(k+1\right) \end{array}\right]}} & = & \underset{A_{i,i}}{\underset{︸}{\left[\begin{array}{cc} A_{p}^{i,i} & O\\ -C_{p}^{i} & I \end{array}\right]}}\underset{x_{i}\left(k\right)}{\underset{︸}{\left[\begin{array}{c} x_{p}^{i}\left(k\right)\\ {\bar{x}}_{p}^{i}\left(k\right) \end{array}\right]}}+\underset{B_{i,i}}{\underset{︸}{\left[\begin{array}{c} B_{p}^{i,i}\\ O \end{array}\right]}}u_{i}\left(k\right)\\ & + & \underset{B_{i,i-1}}{\underset{︸}{\left[\begin{array}{c} B_{p}^{i,i-1}\\ O \end{array}\right]}}u_{i-1}\left(k\right)+\underset{R_{{\mathrm{sp}}_{i}}}{\underset{︸}{\left[\begin{array}{c} O\\ I \end{array}\right]}}r_{i}\left(k\right)\\ y_{i}\left(k\right) & = & \underset{C_{i}}{\underset{︸}{\left[\begin{array}{c} C_{p}^{i}\;O \end{array}\right]}}\underset{x_{i}\left(k\right)}{\underset{︸}{\left[\begin{array}{c} x_{p}^{i}\left(k\right)\\ {\bar{x}}_{p}^{i}\left(k\right) \end{array}\right]}} \end{array}$$

where $r_{i}\left(k\right)$ is the imposed reference value at time *k*. $x_{i}\left(k\right)$, $u_{i}\left(k\right)$ and $y_{i}\left(k\right)$ are the extended state, input and output vectors, respectively. Note that the input uncertainty $w_{i}\left(k\right)$ is defined based on the input vector received from the predecessor $u_{i-1}\left(k\right)$. *I* and *O* are the identity and zero matrix, respectively, each with appropriate dimensions.

Hereafter, each sub-system $S_{i}$, ∀i∈N, will be represented by the compact extended model:

(4) $$\begin{array}{ccc} x_{i}\left(k+1\right) & = & A_{i,i}x_{i}\left(k\right)+B_{i,i}u_{i}\left(k\right)+w_{i}\left(k\right)+R_{{\mathrm{sp}}_{i}}r_{i}\left(k\right)\\ w_{i}\left(k\right) & = & B_{i,i-1}u_{i-1}\left(k\right)\\ y_{i}\left(k\right) & = & C_{i}x_{i}\left(k\right) \end{array}$$

where $A_{i,i}$, $B_{i,i}$, $B_{i,i-1}$, $C_{i}$ and $R_{{\mathrm{sp}}_{i}}$ are matrices with adequate dimensions.

Consider linear inequality constraints for the outputs, inputs and uncertainties defined with:

(5) $$y_{i}\in Y_{i},\;u_{i}\in U_{i},\;w_{i}\in W_{i},\;\forall i\in N$$

where $Y_{i}$, $U_{i}$ and $W_{i}$ are sets defined by linear inequalities.

At every sampling time, each agent $A_{i}$, ∀i∈N, solves a min–max optimization problem, which aims to obtain the minimum optimal input with respect to the maximum level of uncertainty received from its neighbour.

(6) $$\begin{array}{cc} J_{i}\left(x_{i}^{0}\right) & =\min_{\begin{array}{c} u_{i}^{0},\ldots ,u_{i}^{N_{p}-1}\\ {\hat{u}}_{i}^{\max} \end{array}}\max_{w_{i}^{0},\ldots ,w_{i}^{N_{p}-1}}\\ \; & \|R_{w_{i}}{\hat{u}}_{i}^{\max}{\|}_{1}+\sum_{l=0}^{N_{p}-1}\|r_{i}^{l}-y_{i}^{l}{\|}_{1}+\|R_{u_{i}}u_{i}^{l}{\|}_{1}+\|r_{i}^{N_{p}}-y_{i}^{N_{p}}{\|}_{1}\\ s.\;t.\;(4)\\ \; & y_{i}^{l}\in Y_{i},\;l=1,\ldots ,N_{p}-1\\ \; & y_{i}^{N_{p}}\in \Omega_{i}\\ \; & u_{i}^{l}\leq {\hat{u}}_{i}^{\max}\leq u_{i}^{\max},\;l=0,\ldots ,N_{p}-1\\ \; & w_{i}^{l}\leq w_{i}^{\max} \end{array}$$

where $y_{i}^{l}=y_{i}\left(k+l|k\right)$ denotes the output predictions for sub-system $S_{i}$ at time k+l, computed at time step *k*; this is calculated recursively starting from the initial state $x_{i}^{0}=x_{i}\left(k\right)$ measured at time k, using the model (4); the input sequence $u_{i}^{l}=\left\{u_{i}^{0},\ldots ,u_{i}^{N_{p}-1}\right\}$ computed over the prediction horizon $N_{p}$; and the uncertainty sequence $W_{i}=B_{i,i-1}U_{i-1}$ received from the neighbour (where $W_{i}$ is the uncertainty polytope and $U_{i-1}=\{u\in R^{m_{i}}:Au\leq b$ is a H-polytope); $r_{i}^{l}$ is the value at time k+l for the output reference trajectory; $r_{i}^{N_{p}}$ and $y_{i}^{N_{p}}$ are the values for the reference and the output trajectories, at the end of the prediction horizon $k+N_{p}$, respectively; $u_{i}^{\max}$, $w_{i}^{\max}$ are the maximum limits for the input and the uncertainty sequences, respectively; ${\left\|.\right\|}_{1}$ denotes the 1-norm; $R_{u_{i}}\in R^{m_{i}\times m_{i}}$ and $R_{w_{i}}\in R^{m_{i}\times m_{i}}$ are the weight matrices for the input and self-imposed input limit ${\hat{u}}_{i}^{\max}$. The latter is an additional optimization parameter introduced in the local cost function, and its value is communicated at each sampling period to the neighbour. This will guarantee that the uncertainty level received from the neighbour is smaller than this value, without actually transmitting the entire input sequence. The set $\Omega_{i}$ is a robust positive invariant set used to ensure the closed-loop stability of the algorithm by means of the terminal invariant set.

**Remark** **1.** *The uncertainty in each sub-system model refers to the coupling information that must be received from the neighbouring sub-system. Please note that the local optimization problem (6) minimizes the control input, for the worst-case scenario related to uncertainty level received from the predecessor agent. This means that, although unknown, this uncertainty must be bounded to a known value, which is shared between consecutive sub-system. Moreover, this ensures that each local sub-system is prepared for the disturbance signal, which is received via the coupling links.*

Next, some details regarding the computation of the invariant set $\Omega_{i}$, followed by the proposed coalitional DMPC method are given.

### 2.1. Robust Positive Invariant Set Computation

In this sub-section, the details regarding the computation of the robust positive invariant set $\Omega_{i}$, ∀i∈N, which acts as a constraint region for the terminal state $y_{i}^{N_{p}}\in \Omega_{i}$ are presented. To this end, the procedure firstly introduced in [28] is briefly summarized below, tailored for the extended sub-system model.

For each sub-system $S_{i}$, ∀i∈N, with the model defined in (4) and subject to constraints (5), only the nominal model (i.e., $w_{i}$ and $r_{i}$ are zero) is considered. Let us compute a local linear feedback $u_{i}=K_{i}x_{i}$, which ensures that the closed loop eigenvalues are in the unit circle. One suggestion to compute the state feedback matrix $K_{i}$ is to apply classical state-space feedback control designed for the nominal model using Ackermann’s formula (i.e., solving a pole allocation problem) [31], or to calculate it through the minimization of a linear-quadratic cost function, by solving a discrete-time Riccatti Equation [32].

The set $\Omega_{i}$ is robust positive invariant for the nominal model from (4), if the following assumption holds [28,33]:

(7) $$\begin{array}{cc} & x_{i}\in \Omega_{i}\to \left(A_{i,i}+B_{i,i}K_{i}\right)x_{i}+w_{i}\in \Omega_{i},\\ & K_{i}x_{i}\in U_{i},\;C_{i}\Omega_{i}\subseteq Y_{i},\;\forall w_{i}\in W_{i} \end{array}$$

It is worth mentioning the following observations regarding the use of the invariant set in the C-DMPC context:

- the default working framework is non-cooperative DMPC, which implies that each agent $A_{i}$, ∀i∈N, from the multi-agent application communicates with its neighbour, in order to compute the local solution;
- each sub-system model $S_{i}$, ∀i∈N, is subject to input uncertainties received from the sub-system to whom it is connected (in our case its predecessor);
- to provide a simplified algorithm with minimal communication load in the network, only the self-imposed upper bound for the local input trajectory is broadcast in the network (i.e., the optimization variable ${\hat{u}}^{\max}$ introduced in (6));
- a table with different predefined robust positive invariant sets $\Omega_{i}$ is computed using the constraints limits from (5), in which each element is a particular combination of the variable bounds (see Algorithm 1);
- at each sampling period, after the uncertainty upper bound is received from the neighbour, each agent $A_{i}$ uses this information to compute the uncertainty polytope. Next, from the predefined terminal sets table, a set $\Omega_{i}$ is searched for, which includes the received uncertainty polytope (i.e., which will ensure a local feasible solution in the terminal state framework).

Further on, the pseudo-code algorithm used to compute the invariant set table is provided (where for simplicity the sub-system indices are omitted):

Thus, each agent $A_{i}$, ∀i∈N, uses Algorithm 1 in the initialization phase of the proposed method to compute a table of invariant sets $\Omega_{i}$, for different input and uncertainty parametrizations (i.e., distinct combinations for the two parameters α and β). Note that the first set $\Omega_{i}$ from the table corresponds to the largest value for the input constraint, denoted $u^{max}$, whereas the uncertainty has the smallest value. The latter is gradually increased with a step size denoted $step_{\beta }$, until it reaches its maximum admissible value $w^{max}$. In doing so, the size of the invariant set slowly reduces, as the input constraint limit value decreases with a step size denoted $step_{\alpha }$ and the uncertainty level rises.

In practice, a good start for $u^{max}$ and $w^{max}$ bounds are the values for the imposed constraints (5). The values of the step size $step_{\alpha }$, $step_{\beta }$ should be selected such that the table size remains reasonable, with various invariant sets. Moreover, the limits in the state constraints are considered fixed, according to the sub-systems dynamics and used to compute every set $\Omega_{i}$ from the table.
**Algorithm 1****For**$\alpha =u^{max}:-step_{\alpha }:0.1$ **For**
$\beta =0.1:step_{\beta }:w^{max}$
  1. Compute the inequality constraints:  $$\begin{array}{c} A_{u}u\leq \alpha b_{u};\;A_{w}w\leq \beta b_{w};\;A_{x}x\leq b_{x} \end{array}$$
  2. Compute the robust positive-invariant set:  $$\begin{array}{c} \Omega (A,B,K,A_{u},b_{u},A_{w},b_{w},A_{x},b_{x}) \end{array}$$
  3. Save the information α, β, Ω **end**
**end**

### 2.2. Coalitional Distributed Model Predictive Control (C-Dmpc) Methodology

As previously mentioned, what differentiates our proposed coalitional algorithm from the existing works is the flexible framework set for the cyber-physical multi-agent system with a chain architecture. Hence, at each step time, the agents architecture starts as non-cooperative DMPC and will switch to coalitional DMPC (C-DMPC)—when the local feasibility of the interconnected agents is lost. In the C-DMPC framework, the coalition procedure is initialized without a hierarchical level by the local agents with infeasible problems, because due to the coupling links between sub-systems, if not solved, this problem will propagate among neighbouring sub-systems. Using the communication links, these agents share their optimization status with their neighbour, and after that, one of them is randomly selected to start a coalition. Once the coalition procedure is activated, the agents framework changes.

To simplify the design and computational costs, the size of the coalition is increased gradually, if needed. That is, if a coalition of two agents, coupled with the remaining agents from the network, still does not provide feasible solutions for all involved actors, then more work needs to be done. The idea is to first activate all coalitions of two agents, if needed, then the coalitions of three agents, and so on, until in the end, in the extreme case, all the agents are involved in a single coalition. Note that this last case is equivalent to solving a centralized problem for the multi-agent system and will be used in the last resort, if nothing else solved the infeasibility problems that started the coalitional procedure. The reason for this is related with the coalition dynamics (i.e., when two or more agents form a coalition, their respective sub-system models are aggregated and become a single entity). Thus, the number of the optimization variables in a coalition increases with its size, and the local non-cooperative optimization problem becomes a cooperative one inside the coalition. The extreme case of a ‘grand’ coalition between all agents will aggregate all the sub-systems in a single entity (from the control point of view).

#### 2.2.1. Coalition Dynamics

As described before, our C-DMPC algorithm is tailored specifically for cyber-physical multi-agent systems, linked in a unidirectional communication topology. Thus, the coupling information, which is treated as an uncertainty in the local nominal model of each sub-system $S_{i}$, is received from its predecessor sub-system $S_{i-1}$. To minimize the communication burden between consecutive agents, only the self imposed optimization variable ${\hat{u}}^{\max}$ introduced in (6) is broadcast. This value is firstly used to search for an invariant set inside the predefined table, and secondly acts as the uncertainty limit constraint in the local optimization problem. Using this information, the local optimization problem is then solved, and if the solution is infeasible, then the coalition procedure must be started.

Inside a coalition between different consecutive agents, the aim is to solve a cooperative optimization problem; thus the uncertainty variable becomes fully known. Each agent $A_{i}$, ∀i∈N, can form a coalition only with its predecessor, i.e., agent $A_{i-1}$, due to the particular dynamical coupling between their corresponding sub-systems (i.e., linked in a chain). When this occurs, the agents involved will form a compact set denoted generically C. To simplify the notations, the coalition is described without sub-script indices with the following model:

(8) $$\begin{array}{ccc} x_{C}\left(k+1\right) & = & A_{C}x_{C}\left(k\right)+B_{C}u_{C}\left(k\right)+w_{C}\left(k\right)\\ w_{C}\left(k\right) & = & \sum_{j\in N_{C}}B_{C}^{j}u_{j}\left(k\right)\\ y_{C}\left(k\right) & = & C_{C}x_{C}\left(k\right) \end{array}$$

where $x_{C}$ is the state vector of the coalition, $u_{C}$ is the coalition’s input vector, $w_{C}$ is the uncertainty vector of the coalition and $y_{C}$ is output vector for the coalition. All these vectors are composed by aggregating the local vectors corresponding to each sub-system involved in the coalition (e.g., $x_{C}={\left[x_{i}\right]}_{i\in C}$). Moreover, the matrices $A_{C}$, $B_{C}$, $B_{C}^{j}$ and $C_{C}$ are computed according to the aggregation.

The set $N_{C}$ denotes the coalition’s C neighbour, defined as the predecessor sub-system for the sub-systems inside the coalition (e.g., if Agent 2 and 3 form a coalition, then $N_{C}=\left\{1\right\}$, because sub-system 2 is coupled to sub-system 1; thus the coalition in which Agent 2 is involved must receive relevant information from Agent 1, which is outside the coalition and solves a non-cooperative DMPC problem). Moreover, following this reasoning, a coalition involving Agent 1 does not have neighbours (i.e., $N_{C}=\emptyset$, because Agent 1 does not have predecessors).

#### 2.2.2. Coalition Problem Definition

In this section, some details regarding the construction of the constraints sets imposed for the coalition and the optimization problem solved by the coalition are presented.

Hence, the constraint sets for the coalition C are computed as the union of the constraints sets (5) corresponding to each agent $A_{i}$, i∈C:

(9) $$\begin{array}{c} y_{C}\in Y_{C}=\prod_{i\in C}Y_{i},\;\;u_{C}\in U_{C}=\prod_{i\in C}U_{i},\;\;w_{C}\in W_{C}=\prod_{i\in C}W_{i},\;\; \end{array}$$

and the min–max optimization problem solved by the coalition is:

(10) $$\begin{array}{ccc} J_{C}\left(x_{C}^{0}\right) & = & \min_{\begin{array}{c} u_{C}^{0},\ldots ,u_{C}^{N-1}\\ {\hat{u}}_{C}^{\max} \end{array}}\max_{w_{C}^{0},\ldots ,w_{C}^{N-1}}\\ & & \|R_{w_{C}}{\hat{u}}_{C}^{\max}{\|}_{1}+\sum_{l=0}^{N_{p}-1}\|r_{C}^{l}-y_{C}^{l}{\|}_{1}+\|R_{u_{C}}u_{C}^{l}{\|}_{1}+\|r_{C}^{N_{p}}-y_{C}^{N_{p}}{\|}_{1}\\ s.\;t.\;(8)\\ y_{C}^{l} & \in & Y_{C},\;l=1,\ldots ,N_{p}-1\\ y_{C}^{N_{p}} & \in & \Omega_{C}\\ u_{C}^{l} & \leq & {\hat{u}}_{C}^{\max}\leq u_{C}^{\max}\\ w_{C}^{l} & \leq & w_{C}^{\max} \end{array}$$

The weighting matrices $R_{u_{C}}$ and $R_{w_{C}}$ are block diagonal, $\Omega_{C}$ is the aggregated terminal set and $r_{C}^{l}$ and $x_{C}^{0}$ are aggregated vectors containing the corresponding imposed references and initial state values, respectively. $r_{C}^{N_{p}}$ and $y_{C}^{N_{p}}$ are aggregated vectors containing the corresponding imposed references and output predictions values at time $k+N_{p}$, respectively. $u_{C}^{l}$, $w_{C}^{l}$ and $y_{C}^{l}$ are are aggregated vectors containing the corresponding input, uncertainty and output sequences, respectively. ${\hat{u}}_{C}^{\max}$ is an aggregated vector containing the corresponding self-imposed input limits. $u_{C}^{\max}$ and $w_{C}^{\max}$ are aggregated vectors containing the corresponding input and uncertainty limits, respectively.

#### 2.2.3. C-Dmpc Algorithm

To summarize the C-DMPC methodology, the following pseudo-code is provided: With regard to Algorithm 2, the following observations are in order:

- the default uncertainty value used in Step 1 is selected to ensure that optimization problems from Step 3 are feasible, thus ensuring that the proposed methodology is recursively stable (i.e., the terminal set for the coalition is obtained by aggregating the terminal sets of the involved individual agents).
- if the condition from Step 6 is satisfied, then at that sampling period, the working framework is non-cooperative DMPC; otherwise the framework changes to coalitional DMPC (since at least one coalition is activated).
- the priority value, which is used as a condition term to initialize a coalition, is defined by each agent as a random sub-unitary number. In this manner, there is no use of a hierarchical control level to assign these priorities.
- in the extreme, all the agents can be combined in a coalition (C=N), which corresponds to a centralized MPC working framework.
- one or more coalitions can be active simultaneously and are dissolved at the end of each sampling period.

**Remark** **2** ([28]). *In Algorithm 2, the coalitional control problem is feasible (i.e., Step 6. (c). ii.), because $W_{C}\subseteq \prod_{i\in C}W_{i}$, $U_{C}=\prod_{i\in C}U_{i}$ and $\Omega_{C}=\prod_{i\in C}\Omega_{i}$. The stability of the coalition is ensured by the terminal constraint set of the coalition, which is calculated as the Minkowski sum of the terminal sets polytopes defined for each individual agent from the coalition. The coalitional algorithm is recursive-feasible, contingent on Step 3, for which all the optimization problems are feasible, i.e., for which systems can work in a decentralized fashion.*

Next, the C-DMPC methodology is validated in simulation, and the results are provided in Section 3.

## 3. Illustrative Example

In this section, the simulation results and discussion for the C-DMPC method are presented. The proposed simulation scenario for the cyber-physical multi-agent system described in Section 2, Figure 1, has the following characteristics:

- Four heterogeneous discrete-time sub-systems $S_{i}$, ∀i∈{1,…,4}, coupled in a chain architecture were defined using (1), with the following numerical matrices:
  (11) $$\begin{array}{c} S_{1}:A_{p}^{1,1}=\left[\begin{array}{cc} 0.7913 & 0.2020\\ 0.1010 & 0.8417 \end{array}\right]\;B_{p}^{1,1}=\left[\begin{array}{c} 0.0271\\ 0.2291 \end{array}\right]\\ B_{p}^{1,0}=\left[\begin{array}{c} 0\\ 0 \end{array}\right]\;C_{p}^{1}=\left[\begin{array}{cc} 0 & 1 \end{array}\right]\; \end{array}$$
  (12) $$\begin{array}{c} S_{2}:A_{p}^{2,2}=\left[\begin{array}{cc} 0.7936 & 0.1996\\ 0.1198 & 0.8236 \end{array}\right]\;B_{p}^{2,2}=\left[\begin{array}{c} 0.0269\\ 0.2265 \end{array}\right]\\ B_{p}^{2,1}=\left[\begin{array}{c} 0.0004\\ 0.0034 \end{array}\right]\;C_{p}^{2}=\left[\begin{array}{cc} 0 & 1 \end{array}\right]\; \end{array}$$
  (13) $$\begin{array}{c} S_{3}:A_{p}^{3,3}=\left[\begin{array}{cc} 0.7888 & 0.2043\\ 0.0817 & 0.8604 \end{array}\right]\;B_{p}^{3,3}=\left[\begin{array}{c} 0.0273\\ 0.2316 \end{array}\right]\\ B_{p}^{3,2}=\left[\begin{array}{c} 0.0004\\ 0.0034 \end{array}\right]\;C_{p}^{3}=\left[\begin{array}{cc} 0 & 1 \end{array}\right]\; \end{array}$$
  (14) $$\begin{array}{c} S_{4}:A_{p}^{4,4}=\left[\begin{array}{cc} 0.7912 & 0.1994\\ 0.0997 & 0.8211 \end{array}\right]\;B_{p}^{4,4}=\left[\begin{array}{c} 0.0269\\ 0.2263 \end{array}\right]\\ B_{p}^{4,3}=\left[\begin{array}{c} 0.0004\\ 0.0034 \end{array}\right]\;C_{p}^{4}=\left[\begin{array}{cc} 0 & 1 \end{array}\right]\; \end{array}$$
- The limit constraints for the inputs, disturbances and outputs are the following:
  (15) $$\begin{array}{c} \left\{\begin{array}{c} u_{i}^{\min}=-5\\ u_{i}^{\max}=5 \end{array}\right. \end{array}\left\{\begin{array}{c} w_{i}^{\min}=-1\\ w_{i}^{\max}=1 \end{array}\right.\left\{\begin{array}{c} w_{i}^{\min}=-8\\ w_{i}^{\max}=8 \end{array}\begin{array}{c} \forall i\in \{1,\ldots ,4\} \end{array}\right.$$
- For all sub-systems $S_{i}$, ∀i∈{1,…,4}, the following optimization parameters are used: the prediction horizon $N_{p}=5$, the input weights $R_{u_{i}}=0.1$ and $R_{w_{i}}=0.01$.

**Remark** **3.** *The optimization parameters were carefully selected after a thorough analysis from the point of view of achieved performances. Several tests were performed, with different values for the weights and the prediction horizon. The chosen values ensured the best performances.*

- The feedback laws were computed using classical state-feedback control based on the Ackermann’s formula [31], applied for the extended model (4), obtaining:
  (16) $$\begin{array}{c} K_{1}=\left[0.5494\;-2.6061\;0.7488\right],\\ K_{2}=\left[0.3473\;-2.5199\;0.7047\right],\\ K_{3}=\left[0.6249\;-2.6565\;0.7401\right],\\ K_{4}=\left[0.4355\;-2.5107\;0.7051\right]. \end{array}$$

**Remark** **4.** *The Ackermann’s formula [31] was used to achieve specific closed-loop transient performances, chosen as an overshoot value of 5% and settling time of 5 time units, for sub-systems $S_{1}$ and $S_{3}$, and an overshoot value of 4%, and the same settling time, corresponding to sub-systems $S_{2}$ and $S_{4}$. These performance values, were accordingly selected based on each sub-system dynamics.*

- The reference tracking scenario was constructed for 12 time samples, using a sampling period $T_{s}=0.25s$, with the following imposed references:
  (17) $$\begin{array}{c} r_{1}=\left[0.2\;0.2\;0.2\;0.2\;0.2\;0.2\;0.5\;0.5\;0.5\;0.5\;0.5\;0.5\right],\\ r_{2}=\left[0.2\;0.2\;0.2\;0.2\;0.2\;0.2\;0.2\;0.2\;0.2\;0.2\;0.2\;0.2\right],\\ r_{3}=\left[0.2\;0.2\;0.2\;0.2\;0.5\;0.5\;0.5\;0.5\;0.2\;0.2\;0.2\;0.2\right],\\ r_{4}=\left[0.2\;0.2\;0.2\;0.2\;0.2\;0.2\;0.2\;0.2\;0.2\;0.2\;0.2\;0.2\right]. \end{array}$$

Since our proposed scenario has four sub-systems in a chain architecture with unidirectional communication links between the agents, there are eight possible frameworks including coalitions of two, three or four agents defined as follows:

1. default case—no coalitions between $A_{1}$, $A_{2}$, $A_{3}$, $A_{4}$;
2. coalition $C_{12}$ between $A_{1}$ and $A_{2}$, while $A_{3}$, $A_{4}$ remain outside the coalition but interconnected;
3. coalition $C_{123}$ between $A_{1}$, $A_{2}$, and $A_{3}$, while $A_{4}$ remains outside the coalition but interconnected;
4. twosimultaneous active coalitions $C_{12}$ and $C_{34}$ between $A_{1}$ and $A_{2}$ and $A_{3}$ and $A_{4}$, respectively, which are interconnected;
5. coalition $C_{23}$ between $A_{2}$ and $A_{3}$, while $A_{1}$, $A_{4}$ remain outside the coalition but interconnected;
6. coalition $C_{234}$ between $A_{2}$, $A_{3}$ and $A_{4}$, while $A_{1}$ remains outside the coalition but interconnected;
7. coalition $C_{34}$ between $A_{3}$ and $A_{4}$, while $A_{1}$, $A_{2}$ remain outside the coalition but interconnected;
8. extreme case: coalition $C_{1234}$ between all agents $A_{1}$, $A_{2}$, $A_{3}$, $A_{4}$.

**Algorithm 2** Initialization: For each agent $A_{i}$, ∀i∈N, compute a table $T_{i}$, with potential terminal sets $\Omega_{i}$. At each sampling time *k*, each agent $A_{i}$, ∀i∈N, receives the local state value and performs the following steps: 1. Computes the uncertainty polytope using default limit values for the constraints:$$\begin{array}{ccc} W_{i} & = & B_{i,i-1}U_{i-1}^{0}\\ U_{i-1} & = & [u_{i-1}^{\max,0};-u_{i-1}^{\max,0}]. \end{array}$$ 2. Searches in the predefined table $T_{i}$ for a terminal set $\Omega_{i}^{0}$ that includes the default uncertainty $W_{i}\subseteq \Omega_{i}^{0}$. 3. Solves the local optimization problem (6) and obtains the optimal values $U_{i}^{*,0}$, ${\hat{u}}_{i}^{\max,0}$ using the default values $\Omega_{i}=\Omega_{i}^{0}$ for the terminal set and the uncertainty constraint limit ($w_{i}^{\max}=u_{i-1}^{\max,0}$). 4. Broadcasts to its successor the local optimal value ${\hat{u}}_{i}^{\max,0}$ and receives the corresponding value ${\hat{u}}_{i-1}^{\max,0}$ from its predecessor. 5. Repeats Steps 1–3 using the uncertainty constraint value received in Step 4. 6. Checks the feasibility of the local optimization problem: ***If*** the optimization problem from Step 5 is feasible: *then*: Coalitions between agents are not necessary.Each local agent $A_{i}$ sends to its sub-system $S_{i}$, the first value from the optimal trajectory $U_{i}^{*}$; *else*: Coalitions between agents are necessary. In this case, in order to be included in a coalition, each agent $A_{i}$, ∀i∈N, performs the following steps:   a. Receives, from its predecessor, a coalitional report containing the following information: the feasibility status (for the local optimization problem solved at Step 5) and priority value relating to all the predecessor agents from the chain architecture.   b. Sends to its successor, the updated coalitional report (i.e., all the relevant information received, together with its own local feasibility and priority data).   c. Initializes a coalition only if its local priority is the highest from the report. Within a coalition between two agents, the following steps are performed:      i. the coalition model is defined as (8);      ii. the optimization problem (10) subject to (9) is solved.      iii. the relevant information is broadcast to the coalition’s neighbour.      iv. a feasibility check for all the optimization problems is done.       ***If*** the all the optimization problems are feasible:       *then*: The existing coalition was successful and can be dissolved after every sub-system $S_{i}$ receives the first value from the optimal trajectory $U_{i}^{*}$;       *else*: The existing coalition was not successful. Another agent must be included in the existing coalition (if the coalition’s status is infeasible), or another coalition can be activated (if more agents outside the existing coalition have infeasible problems). At this stage, Step (c) is repeated as necessary. 7. End algorithm.

**Remark** **5.** *Please note that our proposed Coalitional DMPC algorithm is tailored specifically for cyber-physical multi-agent systems. The key feature is its capability to switch between control architectures, whenever the feasibility of the multi-agent system is lost, due to uncertainties in the local sub-systems. One example of such multi-agent system is a vehicle platoon. In this case, it is clear that classical centralized MPC is not suitable for controlling this application. Moreover, decentralized MPC, in which the couplings between sub-systems are ignored, can render instability within the platoon. One compromise solution is distributed MPC, in which the interactions are taken into account when computing the local solutions. However, if the distributed MPC (i.e., in non-cooperative framework) fails at this task, then our proposed coalitional DMPC provides a backup plan, namely to merge different sub-systems into coalitions. Inside a coalition, all the information is known; thus, only the coupling signals with sub-systems outside the coalition must be accounted for.*

**Figure 2:**
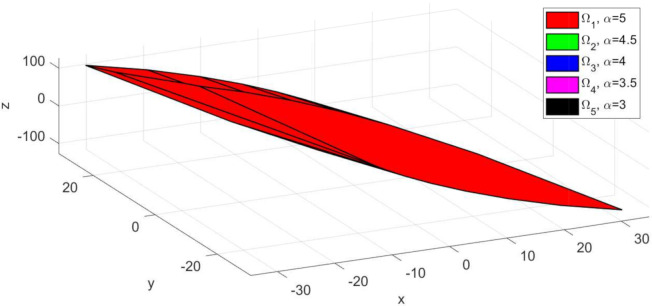
Depiction of the predefined invariant sets corresponding to sub-system S1, computed for uncertainty constraint limit value β=0.1.

**Figure 3:**
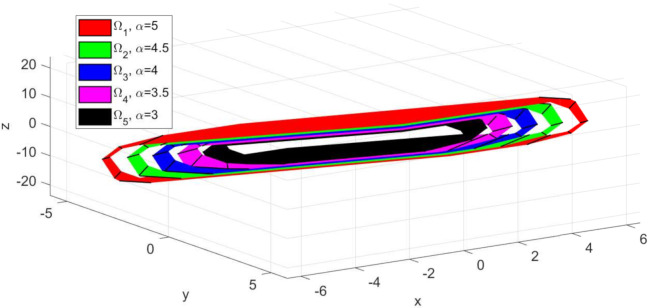
Detail regarding the depiction of the predefined invariant sets corresponding to sub-system S1, computed for uncertainty constraint limit value β=0.1.

The invariant sets obtained for sub-system $S_{1}$ using Algorithm 1 presented in Section 2.1 are depicted in Figure 2. For the computation, the following numerical values were used: $u^{max}=5$, $w^{max}=5$, $step_{\alpha }=step_{\beta }=0.5$. As expected, the larger invariant set (depicted with red colour) was obtained for $\alpha =u^{max}=5$ and β=0.1. Moreover, as the constraint limits become smaller, the set $\Omega_{i}$ decreases in dimension and is included in the larger red set (Figure 3—the sets plotted with green, blue, magenta and black colours, respectively). Since, the state variable for the extended model (4) has three values, the computed invariant sets are three dimensional and can be plotted as convex hulls (ref. Figure 2 and Figure 3). This graphical representation of the invariant sets, which are predefined options for the terminal set constraint (6), are also useful when defining the reference target for the multi-agent system. Thus, one must take into account that the imposed trajectory for each sub-system $S_{i}$, ∀i∈{1,…,4} should be placed in the interior of the invariant set.

**Remark** **6.** *It is worth mentioning that the step values $step_{\alpha }=step_{\beta }=0.5$ were selected taking into account the numerical values of the input and uncertainty constraints to ensure a sufficient number of invariant sets computed. If a smaller value, e.g., $step_{\alpha }=step_{\beta }=0.1$ is chosen, the result would be an increased size for the table containing the invariant sets. However, as depicted in Figure 2, these values also parametrize the dimensions of the invariant set polytopes. Thus, although we would have more available sets, their dimensions would be too similar, to justify the involved computational costs.*

**Figure 4:**
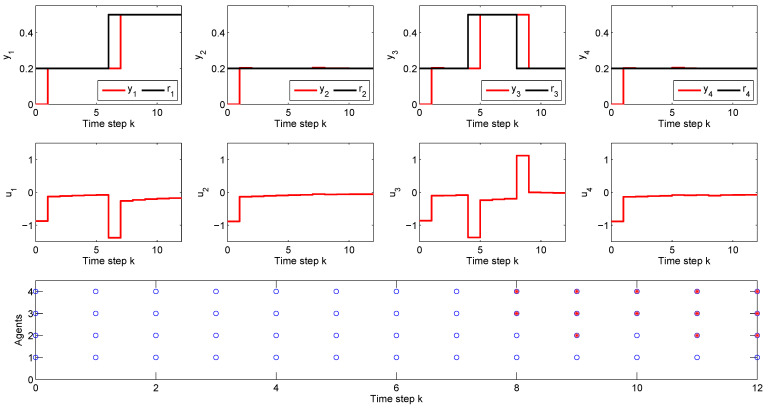
Reference tracking results (subplots 1–4), control efforts (subplots 5–8) and the corresponding coalitions formation (subplot 9) for a cyber-physical multi-agent system composed of 4 interconnected sub-systems.

The reference tracking results and the formation of the coalitions during the simulation are presented in Figure 4.

As depicted in Figure 4, lower subplot, during the first seven time steps, the simulation runs in the default scenario, in which all the agents solve a non-cooperative DMPC algorithm without being involved in a coalition. This is marked with blue circles, at each time step, for each agent $A_{i}$, ∀i∈{1,…,4}. At time step 8, due to the setpoint change of 0.3 units in $r_{3}$ for sub-system $S_{3}$ and the corresponding increase in the control effort $u_{3}$, the local feasibility for sub-system $S_{4}$ is lost. Hence, the coalition $C_{34}$ is activated, which is plotted with a red star marker for $A_{3}$ and $A_{4}$. At the next time step, coalition $C_{234}$ between agents $A_{2}$, $A_{3}$ and $A_{4}$ is active and is coupled with the remaining agent $A_{1}$, because sub-system $S_{2}$ is dynamically coupled through the input with $S_{1}$ and their corresponding agents share information. At time step 10, coalition $C_{34}$ is activated, and for the remaining two time steps of the simulation, coalition $C_{234}$ is active. Moreover, the reference tracking results show that all the imposed set-points are successfully reached in one sampling time, with zero offset error. This occurs for the first seven time steps, in which all the agents work outside a coalition, and also for the remaining simulation time, when coalitions of two or three agents are necessary to maintain the feasibility of the CP-MAS. The results clearly prove the efficiency of our proposed C-DMPC method in a reference tracking scenario.

## 4. Conclusions

In this work, a coalitional distributed model predictive (C-DMPC) methodology suitable for input coupled cyber-physical multi-agent systems was proposed. The algorithm was tailored for an in-chain system architecture with unidirectional communication links (i.e., the coupling information viewed as an uncertainty in the local nominal model was broadcast from a predecessor sub-system to a successor). The methodology was validated in simulation, using an academic cyber-physical multi-agent system as a proof of concept for the proposed algorithm. The simulation results show that if the uncertainty level received by the local agent is manageable, a non-cooperative DMPC algorithm could be locally solved. However, when the local feasibility of the optimization problem was lost, then forming coalitions between agents showed satisfactory performance and the usefulness of the C-DMPC algorithm was proven.

Future work will test the efficiency of the proposed algorithm on a vehicle platooning application.

## 5. Materials and Methods

The simulations from this work were performed using MATLAB R2020b on Windows 10, 64-bit Operating System with a laptop Intel Core i7-9850H CPU @ 2.60 GHz and 16 GB RAM.

The optimizations were implemented using the YALMIP toolbox [34]. 

## Data Availability

Not applicable.

## Abbreviations

The following abbreviations are used in this manuscript:

MPC	Model Predictive Control
DMPC	Distributed Model Predictive Control
C-MPC	Coalitional Model Predictive Control
C-DMPC	Coalitional Distributed Model Predictive Control
CP-MAS	Cyber-Physical Multi-Agent System
CPsS	Cyber-Physical sub-system
